# Epidemic Management via Imperfect Testing: A Multi-criterial Perspective

**DOI:** 10.1007/s11538-023-01172-1

**Published:** 2023-06-09

**Authors:** Giuseppe Palma, Damiano Caprioli, Lorenzo Mari

**Affiliations:** 1 Institute of Nanotechnology, National Research Council, Campus Ecotekne, Via Monteroni, 73100 Lecce, LE Italy; 2 Department of Astronomy & Astrophysics, E. Fermi Institute, University of Chicago, 5640 South Ellis Avenue, Chicago, IL 60637 USA; 3 Dipartimento di Elettronica, Informazione e Bioingegneria, Politecnico di Milano, Via Ponzio 34/5, 20133 Milano, MI Italy

**Keywords:** Compartmental model, Misdiagnosis, Reproduction number, Pareto efficiency, 37N25, 92D30

## Abstract

Diagnostic testing may represent a key component in response to an ongoing epidemic, especially if coupled with containment measures, such as mandatory self-isolation, aimed to prevent infectious individuals from furthering onward transmission while allowing non-infected individuals to go about their lives. However, by its own nature as an imperfect binary classifier, testing can produce false negative or false positive results. Both types of misclassification are problematic: while the former may exacerbate the spread of disease, the latter may result in unnecessary isolation mandates and socioeconomic burden. As clearly shown by the COVID-19 pandemic, achieving adequate protection for both people and society is a crucial, yet highly challenging task that needs to be addressed in managing large-scale epidemic transmission. To explore the trade-offs imposed by diagnostic testing and mandatory isolation as tools for epidemic containment, here we present an extension of the classical Susceptible-Infected-Recovered model that accounts for an additional stratification of the population based on the results of diagnostic testing. We show that, under suitable epidemiological conditions, a careful assessment of testing and isolation protocols can contribute to epidemic containment, even in the presence of false negative/positive results. Also, using a multi-criterial framework, we identify simple, yet Pareto-efficient testing and isolation scenarios that can minimize case count, isolation time, or seek a trade-off solution for these often contrasting epidemic management objectives.

**Supplementary Information:**

The online version contains supplementary material available at 10.1007/s11538-023-01172-1.

## Introduction

With more than 6.9 million confirmed deaths globally, as of April 2023 (World Health Organization 2023), the COVID-19 pandemic ranks as one of the deadliest in history (Piret and Boivin 2021). It has quickly proved to be one of the most socioeconomically devastating too, with ubiquitous social impacts (Saladino et al. 2020) and long-lasting implications for many economic sectors (Nicola et al. 2020). These effects are intensified by the tight interconnectedness of our globalized world, on one hand, and may further contribute to exacerbating rapid patterns of change in the human-Earth system, on the other (Folke et al. 2021). For a large share of the general population, the COVID-19 pandemic has also represented a crash course in epidemiology (Cobey 2020), and created widespread public interest in infectious disease dynamics and epidemic control practice. In particular, it exposed a general audience, in many cases for the first time, to a reflection on the threats posed by emerging and re-emerging diseases (Morens and Fauci 2020), as well as on the complex trade-offs inherent to public health policy (Reed and Gonzalez 2020; Norheim et al. 2021).

Among the difficult positions that governments have been forced to take since the beginning of the COVID-19 pandemic, perhaps none has been more consequential than managing to strike a balance between controlling the spread of the SARS-CoV-2 virus within an initially fully naive population and allowing a basic level of socioeconomic activity. Prior to the development and widespread availability of vaccines (Li et al. 2020), public health interventions were essentially non-pharmaceutical (Ferretti et al. 2020; Flaxman et al. 2020; Hsiang et al. 2020; Kucharski et al. 2020; Lai et al. 2020; Brauner et al. 2021), including both population-wide measures, ranging from social distancing mandates to mobility restrictions and even general lockdowns, and individual prescriptions, like isolation of infected individuals and quarantine for their close contacts. Clearly, the latter family of measures may be less socioeconomically costly than the former, but key to its implementation is the availability of testing tools to effectively identify infection cases. For the COVID-19 pandemic, diagnostic molecular testing was rapidly made possible by the prompt publication of the SARS-CoV-2 genome just weeks into the pandemic; later on, the development of antigen-based lateral flow assays allowed a massive ramping up of surveillance testing (Mercer and Salit 2021; Mina and Andersen 2021). Large-scale application of rapid antigen testing, coupled with contact restrictions, has proved to be highly effective at reducing the prevalence of disease (Pavelka et al. 2021).

Because of its importance for transmission containment, testing has been included in many leading modeling efforts to describe the dynamics of the COVID-19 pandemic (as well as of previous epidemics; see Lipsitch et al. 2003, for a notable precedent concerning the 2002–2004 SARS outbreak) in different spatiotemporal settings (Kraemer et al. 2020; Kucharski et al. 2020; Gatto et al. 2020; Giordano et al. 2020). Some studies have explicitly looked into testing (typically, in association with mandatory isolation of infected individuals) as a tool to possibly control the pandemic (Hellewell et al. 2020; Pettengill and McAdam 2020; Choi and Shim 2021; Wells et al. 2021; Baik et al. 2022), especially after the first national lockdowns started to be lifted (Aleta et al. 2020; Bertuzzo et al. 2020; Di Domenico et al. 2020; Bosetti et al. 2021; Mari et al. 2021; Wang et al. 2022). However, most modeling studies did not explicitly consider that, like all binary classifiers, testing is prone to two types of errors: false negatives (i.e., negative test results in the presence of disease, related to type-II errors in statistics), by which undiagnosed infected individuals are allowed to freely circulate in the community, thereby furthering the spread of disease; and false positives (i.e., positive test results in the absence of disease, related to type-I errors), by which non-infected individuals are isolated from the community, thereby unnecessarily increasing the socioeconomic burden of disease.

In this work, we aim to explore the implications of imperfect testing for the dynamics of an infectious disease spreading in a well-mixed population. A few studies already exist that account for the epidemiological and societal consequences of imperfect testing (Gray et al. 2020; Kasy and Teytelboym 2020; Sasikumar and Varma 2021; Baik et al. 2022; Gharouni et al. 2022), in some cases focusing on false negatives only (Bergstrom et al. 2020; Grassly et al. 2020; Bhattacharyya et al. 2021; Thompson and Wattam 2021; Bhaduri et al. 2022)—which may be an understandable choice given the potential implications of undiagnosed infected individuals as disease spreaders. However, the essentially multi-criterial nature (Ehrgott 2005) of the problem posed by imperfect testing seems to be still under-explored. Specifically, here we use a simple compartmental model to discuss quantitatively the trade-offs that unavoidably emerge while trying to simultaneously minimize the health-related burden of disease, as measured for instance by case count, and the socioeconomic burden associated with control measures, as measured for instance by the time spent in isolation by individuals who have been diagnosed as infected through testing. Multi-criterial analysis has already been proposed as an effective means to evaluate the pros and cons of socioeconomically costly control measures (e.g., national lockdowns, school closures) that are sometimes adopted in large-scale transmission settings, like the COVID-19 pandemic (Kochańczyk and Lipniacki 2021; Lasaulce et al. 2021; Wulkow et al. 2021).

The paper is organized as follows. The structure of the model is presented in Sect. 2. In Sect. 3, we evaluate the effective reproduction number of the model subject to controls and discuss under what conditions testing and isolation alone can actually be used to contain an epidemic, even in the presence of false positives/negatives. Some numerical results are presented in Sect. 4, where we also use the concept of Pareto-efficiency to identify efficient testing and isolation scenarios from a multi-criterial perspective. Finally, the epidemiological and socioeconomic implications of our analysis are discussed in Sect. 5, together with some limitations and possible extensions of our work.

## A SIR Model with Testing and Isolation

A simple, yet effective way to analyze how testing and isolation of individuals identified as infected can affect the dynamics of a directly transmitted infectious disease is using as a starting point a standard SIR model, in which the population is divided into the compartments of susceptible (*S*), infected (*I*), and recovered (*R*) people (Anderson and May 1992). We assume that susceptible individuals become infected and infectious upon contact with an infectious individual, and that clearance of infection results in recovery and permanent (or at least long-lasting) immunity from reinfection. On top of this standard set of hypotheses, we introduce the following assumptions:

- infected individuals develop clinical, yet possibly non-specific manifestations of the disease;
- an individual’s infection status can be assessed through suitable diagnostic tools, but testing is imperfect, i.e., it can produce false negative or false positive results in at least a fraction of cases;
- a positive test result causes the recipient to be identified as infected and consequently isolated from the general community;
- isolation ends after a certain amount of time, provided that the isolated individual also tests negative for the pathogen; and
- individuals released from isolation are no longer subject to testing (i.e., they may have received a so-called immunity passport).

An important corollary to the second assumption above is that testing introduces a further stratification in the population, besides the traditional compartmentalization based on the actual infection status, according to which individuals are assigned to an epidemiological compartment (say, $X \in \{ S, I, R\}$). Specifically, as far as testing is concerned, individuals may fall within one of these three categories: those who have never tested positive, $X_n$; those who have tested positive and are currently isolated, $X_p$; and those who have been released from isolation, $X_c$. Because of imperfect testing, this latter test-based stratification only partially corresponds to the former infection-based compartmentalization; the two stratifications would in fact coincide only in the presence of continuous, universal, perfect testing. A similar, double-stratified approach has been recently proposed by Gharouni et al. (2022) to study the efficacy of testing and isolation as means of epidemic control.

**Fig. 1:**
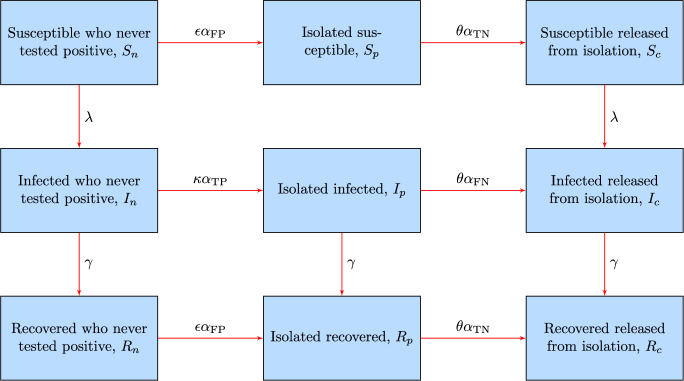
Schematic representation of model (1)

Taken together, the assumptions outlined above translate into the following set of ordinary differential equations (ODEs) describing the dynamics of the abundance (number) of individuals in each of the epidemiological/testing compartments:

1 $$\begin{aligned} \begin{aligned} \dot{S}_n&= - (\lambda + \epsilon \alpha _{\textrm{FP}}) S_n \\ \dot{S}_p&= \epsilon \alpha _{\textrm{FP}} S_n - \theta \alpha _{\textrm{TN}} S_p \\ \dot{S}_c&= \theta \alpha _{\textrm{TN}} S_p - \lambda S_c \\ \dot{I}_n&= \lambda S_n - (\gamma + \kappa \alpha _{\textrm{TP}}) I_n \\ \dot{I}_p&= \kappa \alpha _{\textrm{TP}} I_n - (\gamma + \theta \alpha _{\textrm{FN}}) I_p \\ \dot{I}_c&= \lambda S_c + \theta \alpha _{\textrm{FN}} I_p - \gamma I_c \\ \dot{R}_n&= \gamma I_n - \epsilon \alpha _{\textrm{FP}} R_n \\ \dot{R}_p&= \gamma I_p + \epsilon \alpha _{\textrm{FP}} R_n - \theta \alpha _{\textrm{TN}} R_p \\ \dot{R}_c&= \gamma I_c + \theta \alpha _{\textrm{TN}} R_p \, , \end{aligned} \end{aligned}$$

with

2 $$\begin{aligned} \lambda = \beta \frac{I_n + I_c}{S_n + S_c + I_n + I_c + R_n + R_c} \end{aligned}$$

being the force of infection, described as frequency-dependent. This choice stems from the assumption of a constant contact rate, resulting in an infection rate that depends upon the prevalence (i.e., the frequency) of infectious individuals within the population (Anderson and May 1992). Vital dynamics (birth and death processes) and containment measures other than testing and isolation (e.g., transmission reduction via social distancing and/or adoption of personal protective equipment) are neglected in Eqs. (1) and (2) for the sake of minimality. A schematic representation of the model is shown in Fig. 1.

In the model, $\beta$ and $\gamma$ are the transmission and recovery rates (the only two epidemiological parameters of the standard SIR model; note that we assume that isolation is completely effective at preventing transmission and that recovery rates are independent of the “testing history” of an individual—both of which clearly represent simplifications of the problem at hand), $\epsilon$ and $\kappa$ are the testing rates for non-infected (susceptible and recovered) and infected individuals, $\theta$ is the isolation release rate, which can be thought of as the inverse of the average duration of the isolation order, $\alpha _{\textrm{TP}}$ is the true positive rate (the probability that the test correctly identifies an infected individual, also known as *sensitivity*), $\alpha _{\textrm{FN}} = 1 - \alpha _{\textrm{TP}}$ is the false negative rate (the probability that the test classifies an infected individual as non-infected), $\alpha _{\textrm{TN}}$ is the true negative rate (the probability that the test correctly identifies a non-infected individual, also known as *specificity*), and $\alpha _{\textrm{FP}} = 1 - \alpha _{\textrm{TN}}$ is the false positive rate (the probability that the test classifies a non-infected individual as infected).

Regarding testing, we typically expect $\epsilon \le \kappa$ because of the assumption of infected individuals developing clinical, yet possibly non-specific symptoms of the disease, which could warrant the execution of diagnostic testing; on the other hand, non-infected individuals could still be involved in routine screening, which will occur, in all likelihood, at a lower rate. Introducing such a distinction between testing rates requires neither knowing the infection status of an individual in advance nor defining the testing rates as independent decision variables. Rather, it allows describing in a simple, yet general way the different average probability per unit of time that individuals in different epidemiological compartments may undergo testing based, e.g., on their different likelihood to develop possibly revealing symptoms. For this reason, it has already been widely used in the literature: for instance, the models described in Bertuzzo et al. (2020), Grassly et al. (2020), Gray et al. (2020), Choi and Shim (2021), Mari et al. (2021), Thompson and Wattam (2021), Baik et al. (2022), Gharouni et al. (2022), and Wang et al. (2022) all contemplate some variations of the hypothesis that different testing rates apply to infected vs. non-infected individuals. By contrast, the assumption $\epsilon = \kappa$ is typically done when focusing on generalized mass testing as a means of community screening (see, e.g., Bosetti et al. 2021; Pavelka et al. 2021; Baik et al. 2022; Zhang and Britton 2022) and can obviously be accommodated within our general model as a particular case.

**Fig. 2:**
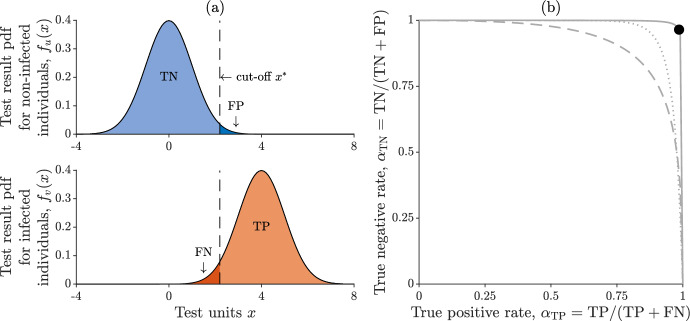
Relationship between false positives and false negatives in test results. **a** Hypothetical probability density functions (pdfs) of testing results for non-infected (top) and infected (bottom) individuals. Both pdfs are assumed to be normal ($f_u(x) = \mathcal {N}(0,1)$, $f_v(x) = \mathcal {N}(4,1)$; *x* is expressed in arbitrary test units). **b** Functional relationship between $\alpha _{\textrm{TN}}$ and $\alpha _{\textrm{TP}}$ (eqn. (3)). The solid curve is obtained with the parameters used in panel a ($a = 4$, $b = 1$) for different values of the cut-off threshold (the black dot corresponds to the cut-off $x^* = 2.2$). The dashed and dotted curves are obtained with $a = 2$, $b = 1$ and $a = 4$, $b = 2$, respectively

Another key aspect of testing is that $\alpha _{\textrm{TP}}$ and $\alpha _{\textrm{TN}}$ are typically not independent of each other. To simply show this, let the test results for non-infected and infected individuals be drawn from two continuous probability distributions $f_u(x)$ and $f_v(x)$, respectively, with *x* representing the variable that is assessed by the test (Fig. 2a). A good test is one for which the overlap of the two distributions is minimal. No matter how good a test is, though, some overlap will practically be unavoidable, and setting a cut-off threshold to separate negative results from positive results will always cause some instances to be misclassified. Indeed, how to optimally select a cut-off is one of the burning questions in the literature on diagnostic testing (e.g., López-Ratón et al. 2014). Specifically, a higher threshold will yield more true negatives (TNs) and fewer false positives (FPs), but also fewer true positives (TPs) and more false negatives (FNs)—and vice versa. Mathematically, if we define $\alpha _{\textrm{TN}} = \textrm{TN} / (\textrm{TN} + \textrm{FP})$ and $\alpha _{\textrm{TP}} = \textrm{TP} / (\textrm{TP} + \textrm{FN})$, then for a given cut-off $x^*$ we get

$$\begin{aligned} \begin{aligned} \alpha _{\textrm{TN}}(x^*)&= F_u(x^*) \\ \alpha _{\textrm{TP}}(x^*)&= 1 - F_v(x^*) \,, \end{aligned} \end{aligned}$$

where $F_u(x)$ and $F_v(x)$ are the cumulative distribution functions of $f_u(x)$ and $f_v(x)$, respectively. Hence,

$$\begin{aligned} \alpha _{\textrm{TN}}(\alpha _{\textrm{TP}}) = F_u \left[ F_v^{-1}(1 - \alpha _{\textrm{TP}}) \right] \,, \end{aligned}$$

or, equivalently,

$$\begin{aligned} \alpha _{\textrm{TP}}(\alpha _{\textrm{TN}}) = 1 - F_v \left[ F_u^{-1}(\alpha _{\textrm{TN}}) \right] \,. \end{aligned}$$

For the sake of concreteness, let $f_u(x)$ and $f_v(x)$ be two normal distributions with assigned means ($\mu _u$ and $\mu _v$) and standard deviations ($\sigma _u$ and $\sigma _v$), i.e., $f_u = \mathcal {N} (\mu _u, \sigma _u)$ and $f_v = \mathcal {N} (\mu _v, \sigma _v)$. With straightforward algebraic manipulations, we get

3 $$\begin{aligned} \alpha _{\textrm{TN}}(\alpha _{\textrm{TP}}) = \Phi [a - b \Phi ^{-1} (\alpha _{\textrm{TP}})] \, , \end{aligned}$$

where $a = (\mu _v - \mu _u) / \sigma _u$, $b = \sigma _v / \sigma _u$, and $\Phi (x)$ is the cumulative distribution function of the standard normal distribution $\mathcal {N}(0,1)$. The functional relationship between $\alpha _{\textrm{TP}}$ and $\alpha _{\textrm{TN}}$ is shown in Fig. 2b for different choices of the parameters *a* and *b*.

## Basic and Effective Reproduction Numbers

To assess the long-term transmission potential of the pathogen, it is useful to evaluate two key epidemiological indexes, namely the basic reproduction number, $\mathcal {R}_0$, and the effective reproduction number, $\mathcal {R}_t$ (Anderson and May 1992; Brauer 2008). These two quantities can be intuitively understood as the average number of secondary infections produced by one infected individual in a completely susceptible population in the absence of controls (including testing and isolation of individuals following a positive test result, $\mathcal {R}_0$) or in a population with prior exposure to the pathogen and/or in the presence of controls ($\mathcal {R}_t$).

### Basic Reproduction Number

If no containment measures are in place (which, for the problem at hand, means that testing and isolation are not enforced), model (1) reduces to the standard SIR model with frequency-dependent transmission and no vital dynamics, for which $\mathcal {R}_0 = \beta / \gamma$ (Anderson and May 1992).

### Effective Reproduction Number

The evaluation of $\mathcal {R}_t$ requires following a specific trajectory in the state space diverging from a given equilibrium condition. We assume that, between the initial time $t = t_0$ and some later time $t = t_{\tau }$, when the enforcement of containment measures begins, the pathogen is left free to invade uncontrolled (and possibly unnoticed) a community that is, at least initially, fully susceptible (and obviously untested). We thus start from an impulsive perturbation of the disease-free equilibrium ${\textbf{x}}_{\textbf{0}} = [N, 0, 0, 0, 0, 0, 0, 0, 0]^T$, where *N* is the total population abundance and the superscript *T* denotes matrix transposition. In this time interval, any changes in $\mathcal {R}_t$ are to be ascribed solely to changes in the state variables—most prominently, the progressive erosion of the susceptible compartment as a result of the unfolding outbreak. After $t = t_{\tau }$, when control measures begin to be applied, the temporal evolution of $\mathcal {R}_t$ reflects both the further changes of the state variables in response to epidemic dynamics and the impact of controls.

Following Diekmann et al. (2010), the evaluation of reproduction numbers from compartmental epidemiological models requires, first, isolating the *infected subsystem*, defined as the set of equations that describe the production of new infections and the state changes of infected individuals. For model (1) with no testing ($\epsilon = \kappa = 0$), the infected subsystem simply corresponds to the equation describing the dynamics of infected individuals in the community, $I_n$, whose linearization gives

$$\begin{aligned} \frac{\partial (\dot{I}_n)}{\partial {I_n}} = \frac{\partial (\lambda S_n)}{\partial {I_n}} - \gamma = \beta \frac{S_n(t) [S_n(t) + R_n(t)]}{N^2} - \gamma \,. \end{aligned}$$

An outbreak will keep on unfolding only if $\partial (\dot{I}_n) /\partial {I_n} > 0$, corresponding to the condition

$$\begin{aligned} \mathcal {R}_t = \frac{\beta }{\gamma } \frac{S_n(t) [S_n(t) + R_n(t)]}{N^2} > 1 \,, \end{aligned}$$

with $\mathcal {R}_t$ being the effective reproduction number evaluated over $t_0 \le t < t_{\tau }$. Note that $\mathcal {R}_t \equiv \mathcal {R}_0$ in a neighborhood of ${\textbf{x}}_{\textbf{0}}$ (fully susceptible population), i.e., prior to the start of the epidemic.

As a result of the implementation of testing at $t = t_{\tau }$, the infected system becomes three-dimensional, now including the infected who never tested positive, $I_n$, those who have been correctly identified through testing and are currently subject to isolation, $I_p$, and those who have been released from isolation before clearing the infection, $I_c$. The dynamics of the infectious subsystem are described by the reduced-order, time-varying Jacobian

$$\begin{aligned} {\textbf{J}}^{*}(t) = \begin{bmatrix} \beta S_n(t) \mathcal {F}(t) - (\gamma + \kappa \alpha _{\textrm{TP}}) &{} 0 &{} \beta S_n(t) \mathcal {F}(t) \\ \kappa \alpha _{\textrm{TP}} &{} - (\gamma + \theta \alpha _{\textrm{FN}}) &{} 0 \\ \beta S_c(t) \mathcal {F}(t) &{} \theta \alpha _{\textrm{FN}} &{} \beta S_c(t) \mathcal {F}(t) - \gamma \end{bmatrix} \,, \end{aligned}$$

with

$$\begin{aligned} \mathcal {F}(t) = \frac{S_n(t) + S_c(t) + R_n(t) + R_c(t)}{[S_n(t) + S_c(t) + I_n(t) + I_c(t) + R_n(t) + R_c(t)]^2} \,. \end{aligned}$$

To evaluate $\mathcal {R}_t$, we apply a next-generation matrix (NGM) approach (Diekmann et al. 1990; Van den Driessche and Watmough 2002) and decompose ${\textbf{J}}^{*}(t)$ into a time-varying transmission matrix

$$\begin{aligned} {\textbf{T}}(t) = \beta \mathcal {F}(t) \begin{bmatrix} S_n(t) &{} 0 &{} S_n(t) \\ 0 &{} 0 &{} 0 \\ S_c(t) &{} 0 &{} S_c(t) \end{bmatrix} \end{aligned}$$

and a transition matrix

$$\begin{aligned} \varvec{\Sigma } = \begin{bmatrix} - (\gamma + \kappa \alpha _{\textrm{TP}}) &{} 0 &{} 0 \\ \kappa \alpha _{\textrm{TP}} &{} - (\gamma + \theta \alpha _{\textrm{FN}}) &{} 0 \\ 0 &{} \theta \alpha _{\textrm{FN}} &{} - \gamma \end{bmatrix} \,, \end{aligned}$$

so that ${\textbf{J}}^{*}(t) = {\textbf{T}}(t) + \varvec{\Sigma }$. The time-varying NGM can then be found as

$$\begin{aligned} \begin{aligned} {\textbf{K}}(t)&= - {\textbf{T}}(t) \varvec{\Sigma }^{-1} = \\&= - \beta \mathcal {F}(t) \begin{bmatrix} S_n(t) &{} 0 &{} S_n(t) \\ 0 &{} 0 &{} 0 \\ S_c(t) &{} 0 &{} S_c(t) \end{bmatrix} \begin{bmatrix} - \frac{1}{\gamma + \kappa \alpha _{\textrm{TP}}} &{} 0 &{} 0 \\ - \frac{\kappa \alpha _{\textrm{TP}}}{(\gamma + \kappa \alpha _{\textrm{TP}}) (\gamma + \theta \alpha _{\textrm{FN}})} &{} - \frac{1}{\gamma + \theta \alpha _{\textrm{FN}}} &{} 0 \\ - \frac{\kappa \alpha _{\textrm{TP}} \theta \alpha _{\textrm{FN}}}{\gamma (\gamma + \kappa \alpha _{\textrm{TP}}) (\gamma + \theta \alpha _{\textrm{FN}})} &{} - \frac{\theta \alpha _{\textrm{FN}}}{\gamma (\gamma + \theta \alpha _{\textrm{FN}})} &{} - \frac{1}{\gamma } \end{bmatrix} = \\&= \frac{\beta }{\gamma } \mathcal {F}(t) \begin{bmatrix} \frac{\gamma (\gamma + \theta \alpha _{\textrm{FN}}) + \kappa \alpha _{\textrm{TP}} \theta \alpha _{\textrm{FN}}}{(\gamma + \kappa \alpha _{\textrm{TP}}) (\gamma + \theta \alpha _{\textrm{FN}})} S_n(t) &{} \frac{\theta \alpha _{\textrm{FN}}}{\gamma + \theta \alpha _{\textrm{FN}}} S_n(t) &{} S_n(t) \\ 0 &{} 0 &{} 0 \\ \frac{\gamma (\gamma + \theta \alpha _{\textrm{FN}}) + \kappa \alpha _{\textrm{TP}} \theta \alpha _{\textrm{FN}}}{(\gamma + \kappa \alpha _{\textrm{TP}}) (\gamma + \theta \alpha _{\textrm{FN}})} S_c(t) &{} \frac{\theta \alpha _{\textrm{FN}}}{\gamma + \theta \alpha _{\textrm{FN}}} S_c(t) &{} S_c(t) \end{bmatrix} \,. \end{aligned} \end{aligned}$$

Interestingly, the elements of the NGM lend themselves to a rather intuitive epidemiological interpretation, as they represent the contributions of a cohort of infected individuals (in the three isolation-related stages, columns) to a new generation of infections (in the three isolation-related stages, rows). Specifically, susceptible individuals (who either have never tested positive, first row, or have been released from isolation, third row) can be infected upon contact with infected individuals who: (first column) have not tested positive yet or tested positive and were released from isolation before recovering after a false-negative test result; (second column) are isolated but get released before recovering after a false-negative test result; or (third column) are in the community after being released from a prior isolation mandate. The effective reproduction number is the spectral radius of the NGM, i.e.,

4 $$\begin{aligned} \mathcal {R}_t = \frac{\beta }{\gamma } \mathcal {F}(t) \frac{S_n(t) [\gamma (\gamma + \theta \alpha _{\textrm{FN}}) + \kappa \alpha _{\textrm{TP}} \theta \alpha _{\textrm{FN}}] + S_c(t) (\gamma + \kappa \alpha _{\textrm{TP}}) (\gamma + \theta \alpha _{\textrm{FN}})}{(\gamma + \kappa \alpha _{\textrm{TP}}) (\gamma + \theta \alpha _{\textrm{FN}})} \end{aligned}$$

for $t \ge t_{\tau }$. From this expression, it is immediate to see that, in the absence of testing (specifically, with $\kappa = 0$, resulting in $S_c(t) = I_c(t) = R_c(t) = 0$), the effective reproduction number just derived reduces to the expression found for $t_0 \le t < t_{\tau }$.

The testing effort that is necessary to asymptotically halt the spread of the pathogen (obviously, with $\mathcal {R}_0 > 1$) can be evaluated from eqn. (4) by calculating the values of the control parameters (in this case, for instance, the infected testing rate $\kappa$) for which the condition $\mathcal {R}_t < 1$ is verified for some *t*. To make the analytical computation easier, we can introduce the simplifying hypothesis that controls are in place from the very beginning of an outbreak ($t_0 \equiv t_{\tau }$). In this case, in a neighborhood of ${\textbf{x}}_{\textbf{0}}$ (fully susceptible and untested population), the effective reproduction number reduces to the so-called control reproduction number (Anderson and May 1992; Brauer 2008), which for the problem at hand reads

$$\begin{aligned} \mathcal {R}_C = \frac{\beta }{\gamma } \frac{\gamma (\gamma + \theta \alpha _{\textrm{FN}}) + \kappa \alpha _{\textrm{TP}} \theta \alpha _{\textrm{FN}}}{(\gamma + \kappa \alpha _{\textrm{TP}}) (\gamma + \theta \alpha _{\textrm{FN}})} \,. \end{aligned}$$

To prevent the long-term circulation of the pathogen through testing and isolation, the condition $\mathcal {R}_C < 1$ must be met. We preliminary note that, for large values of $\kappa$, $\mathcal {R}_C$ tends asymptotically to $(\beta / \gamma ) (\theta \alpha _{\textrm{FN}}) / (\gamma + \theta \alpha _{\textrm{FN}})$, which is larger than one if $\mathcal {R}_0 > \mathcal {R}_0^* = 1 + \gamma / (\theta \alpha _{\textrm{FN}})$. In this case, testing and isolation cannot but fail as the sole means of controlling the outbreak, and other complementary measures must be implemented. Otherwise, for $1< \mathcal {R}_0 < \mathcal {R}_0^*$, testing and isolation can be used to halt disease transmission, provided that testing effort is strong enough, namely if

$$\begin{aligned} \kappa > \frac{\gamma \left( \frac{\beta }{\gamma } - 1 \right) (\gamma + \theta \alpha _{\textrm{FN}})}{\alpha _{\textrm{TP}} \left[ \gamma - \left( \frac{\beta }{\gamma } - 1 \right) \theta \alpha _{\textrm{FN}} \right] } \,. \end{aligned}$$

We note that the right-hand side of the above inequality decreases for decreasing values of $\alpha _{\textrm{FN}}$ and increasing values of $\alpha _{\textrm{TP}}$. In other words, while imperfect testing can curb the spread of disease (under suitable $\mathcal {R}_0$ conditions), better testing can reduce the effort needed to accomplish the goal.

## Numerical Results

Similarly to the evaluation of the effective reproduction number, simulating model (1) also requires a two-step algorithm. In step 1, the model is run with no control measures (i.e., with $\kappa = \epsilon = 0$) over the timespan $t_0 = 0 \le t < t_{\tau }$, with initial conditions $S_n(0) = N - I_0$, $I_n(0) = I_0$, and all other state variables set to zero. In step 2, the model is run including testing and isolation ($\kappa > 0$, $\epsilon > 0$) over the timespan $t \ge t_{\tau }$ and initial conditions taken from the final state of the simulation performed in the first step.

### Numerical Simulation of the Model

**Fig. 3:**
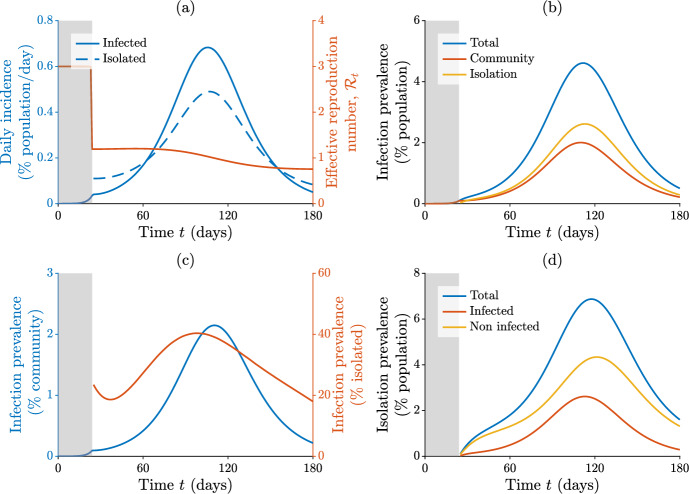
Numerical simulation of model (1). **a** Total number of new daily cases and isolation mandates, expressed as a percentage of the total population (left axis), and effective reproduction number (right axis). **b** Total infection prevalence and breakdown by sub-population (infected individuals in the community or isolated), expressed as a percentage of the total population. **c** Infection prevalence in the community (left axis) and among isolated individuals (right axis); the latter can be evaluated only after the introduction of controls. **d** Total prevalence of isolated individuals and breakdown by actual infection status, expressed as a percentage of the total population. Parameter values: $N = 10^6$, $\beta = 0.429$ day$^{-1}$, $\gamma = 1/7$ day$^{-1}$ (so that $\mathcal {R}_0 = \beta / \gamma = 3$), $\kappa = 1/3$ day$^{-1}$, $\epsilon = 1/60$ day$^{-1}$, $\theta = 1/10$ day$^{-1}$, $\alpha _{\textrm{TP}} = 0.85$, $a = 2.681$, $b = 1$ (so that, using eqn. 3, $\alpha _{\textrm{TN}} = 0.95$). The simulation has been initialized at time $t_0 = 0$ with a seed of $I_0 = 1$ infected individual. Testing and isolation are enforced when the total number of new infections in the previous seven days exceeds a threshold of 100 cases per 100,000 population, which occurs at $t_{\tau } = 24$ days in this simulation (the gray shaded area in each panel thus corresponds to the period of uncontrolled disease transmission). Note that the time series of the isolation-related quantities start from positive values at $t = t_{\tau }$ because all the influxes to the isolated compartments are finite and positive from the beginning of testing owing to the previous uncontrolled spread of the pathogen (Color figure online)

A numerical simulation of model (1) is shown in Fig. 3. The pathogen initially spreads uncontrolled, with a basic reproduction number $\mathcal {R}_0 = 3$ (see the figure caption for the full list of parameters), leading to an exponential growth of infections. We assume that testing and isolation begin to be enforced when the total number of new infections exceeds a threshold of 100 cases per 100,000 population in the previous seven days. In this example, controls are introduced at day $t_{\tau } = 24$, resulting in a $\approx 60 \%$ drop in the effective reproduction number (a). The control effort simulated here is not sufficient to bring $\mathcal {R}_t$ below the critical unit threshold, though, and the epidemic keeps on spreading, albeit more slowly than in a no-control scenario. As a result, $\approx 13\%$ of the population is projected to contract the disease over a time window of $t_{\omega } = 90$ days starting immediately after the introduction of controls, which still represents a $\approx 69 \%$ reduction of the total number of cases expected without controls. Note that case reduction does not entirely correspond to case avoidance: some infections may be delayed rather than prevented, as the epidemic curve is flattened but still increasing. At the peak of the outbreak, $\approx 0.7 \%$ of the population is infected each day (compared to $\approx 6 \%$ without controls), while $\approx 0.5 \%$ enters isolation daily (a). The total prevalence of infection reaches a peak value of $\approx 4.6 \%$ of the population, with more than $50 \%$ of the active cases being subject to isolation throughout the period in which controls are in place (b). Infection prevalence in the community attains a peak value of $\approx 2.1 \%$, while reaching $\approx 40 \%$ among isolated individuals (c). The latter figure suggests that, even at the height of the outbreak, more than half of the isolated individuals are in fact non-infected (because they either have received a false-positive result or are waiting to be released after recovery). Indeed, at peak, $\approx 7 \%$ of the population is subject to an isolation mandate, but only $\approx 2.5 \%$ is actually infected (d). Isolated individuals account for $\approx 2.9 \%$ of the total person-time in the time interval between $t_{\tau }$ and $t_{\tau } + t_{\omega }$ (corresponding to $N t_{\omega }$ person-days), with superfluously isolated individuals (non-infected people subject to isolation) corresponding to $\approx 64 \%$ of this figure (i.e., $\approx 1.9 \%$ of total person-time). Note that the total/superfluous isolation person-time is evaluated as the area below the blue/yellow curve in Fig. 3d between $t = t_{\tau }$ and $t = t_{\tau } + t_{\omega }$.

### Evaluating Testing and Isolation as Containment Tools

**Fig. 4:**
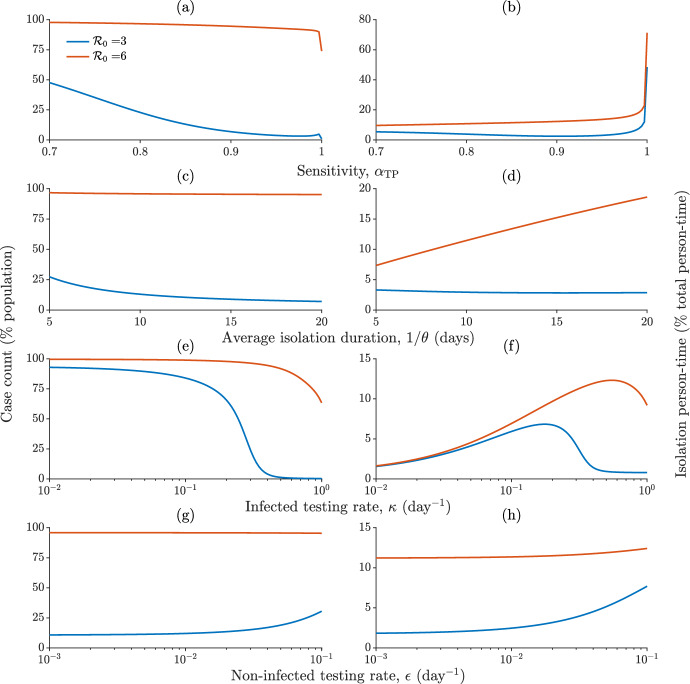
Sensitivity analysis of model (1) with respect to variations of testing and isolation parameters. **a**–**b** Effect of different test sensitivity levels on case count (relative to total population size, a) and isolation person-time (relative to total person-time, b); a timespan of $t_{\omega } = 90$ days after the start of containment measures has been considered to evaluate both indicators. **c**–**h** As in (**a**–**b**), for different average duration of the isolation order (**c**–**d**), testing rates for infected individuals (**e**–**f**), and testing rates for non-infected individuals (**g**–**h**). Parameters and other details as in Fig. 3, except for the scenario with $\mathcal {R}_0 = 6$, which has been obtained by doubling the transmission rate $\beta$ used in the base case $\mathcal {R}_0 = 3$ (Color figure online)

The results shown in Fig. 3 clearly suggest the importance of a careful evaluation of testing and isolation protocols. It could be argued, in fact, that too little testing may do nothing to curb transmission; however, too much *imperfect* testing (namely, with inadequate specificity) may represent an additional socioeconomic threat to a population already burdened by an ongoing epidemic. These insights can be quantitatively corroborated by performing a sensitivity analysis of the model results with respect to variations of the parameters related to testing and isolation, as shown in Fig. 4 for two different values of $\mathcal {R}_0$. Specifically, we focus on two key performance indicators evaluated over a finite period of time after the implementation of controls:

- health-related burden, evaluated as case count, and
- socioeconomic burden, evaluated as person-time spent in isolation.

In the presence of an aggressive pathogen (higher $\mathcal {R}_0$), higher values of test sensitivity (i.e., associated with diagnostic tools with better ability to correctly identify infected individuals) are linked to monotonically declining case count (a); however, the case-count decline remains marginal up to a high sensitivity level ($\alpha _{\textrm{TP}} > 0.99$). With a less aggressive pathogen (lower $\mathcal {R}_0$), instead, the relationship between test sensitivity and case count is monotonically decreasing only up to $\alpha _{\textrm{TP}} \approx 0.98$, after which case count increases, reaching a (small) local maximum before eventually declining again as $\alpha _{\textrm{TP}}$ approaches one. The relationship between sensitivity and isolation person-time is not trivial either (b): for an aggressive pathogen, it is monotonically increasing; for a milder one, a local minimum is projected to occur for $\alpha _{\textrm{TP}} \approx 0.90$. These complex outcomes may be imputed to the link between sensitivity and specificity: as the former increases, the latter decreases, leading to larger fractions of non-infected individuals being isolated and, as a consequence, to higher values of the force of infection in the community. A longer duration of the isolation period determines a monotonic decline of case count (c), as a result of reduced risk of allowing back into the community individuals who are still infectious; however, the case-count decline is negligible with an aggressive pathogen. In this case, longer isolation is associated with a marked increase of isolation person-time, while a small decline of isolation person-time is observed for longer isolation in the presence of a milder pathogen (d). Increasing testing rates for infected individuals may lead to a strong decrease in case count, especially for a milder pathogen (e). On the other hand, the relationship between the testing rate and isolation person-time is nontrivial, peaking at intermediate values in both of the considered $\mathcal {R}_0$ scenarios (f). Higher rates of testing for non-infected individuals may lead to different outcomes in terms of case count depending on the value of $\mathcal {R}_0$ (g): with a more aggressive pathogen, the frequency of testing does not basically influence the case count; with a less aggressive one, more frequent testing leads to a higher case count. The latter, quite unexpected result mainly stems from the nonlinearity of the force of infection (for high values of $\epsilon$, the misclassification of relatively many non-infected individuals determines a reduction in the number of susceptible and recovered individuals in the community—hence a reduction in the denominator of Eq. (2) and, in turn, an increase in the force of infection, yielding a higher case count) and the assumption that individuals released from isolation are no longer subject to testing (for high values of $\epsilon$, relatively many susceptible individuals would falsely test positive, only to be later released in the community where they can get infected and contribute to the spread of disease without being subject to further scrutiny). Finally, higher testing rates for non-infected individuals lead to increasing shares of person-time spent in isolation (h).

### Efficient Testing and Isolation Scenarios

One interesting result emerging from Fig. 4 is the existence of trade-offs between the two selected key epidemiological indicators, namely case count and isolation person-time. If we set these two quantities as the objective functions to be simultaneously minimized while managing an epidemic outbreak, we can apply tools from multi-criterial analysis (Ehrgott 2005) to identify scenarios that efficiently reduce both the health-related and the socioeconomic burden of disease.

**Fig. 5:**
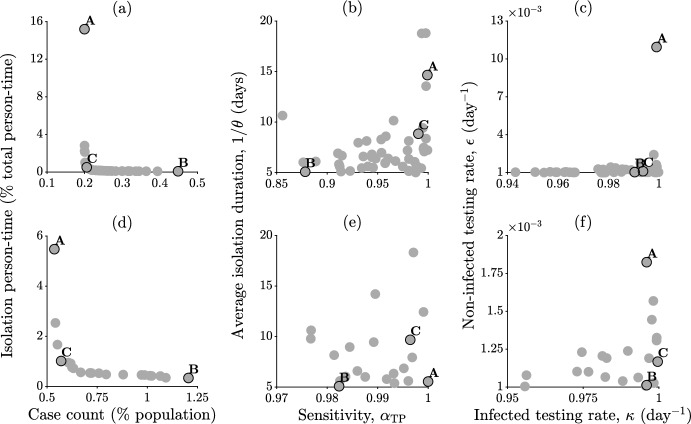
Pareto-efficient scenarios for epidemic control. **a** Pareto front obtained for various combinations of test sensitivity, isolation duration, and testing frequency for infected and non-infected individuals, assuming $\mathcal {R}_0 = 3$. **b**–**c** Two possible projections of the four-dimensional set of Pareto-efficient solutions. **d**–**f** As in a–c, assuming $\mathcal {R}_0 = 6$. Testing and isolation scenarios have been obtained via Latin hypercube sampling ($10^6$ samples) of the parameter space explored in Fig. 4. The points marked as A and B correspond to the solutions with minimum or maximum case counts among the explored alternatives, respectively, while C represents the solution that is closest to the ideal point, where the two objectives are independently minimized. Unspecified parameters and other simulation details as in Fig. 3

Figure 5 shows the two-dimensional Pareto fronts (in the plane of the objective functions) and the corresponding Pareto-efficient solutions (in two different planar projections of the four-dimensional parameter space explored in the sensitivity analysis) for this optimization problem evaluated over the same parameter ranges explored in Fig. 4. As a reminder, given a multi-objective decision problem, the Pareto front is the set of all non-dominated solutions, which in turn are defined as those alternatives that cannot be perturbed without resulting in a worsening of at least one of the objectives. The Pareto front obtained for $\mathcal {R}_0 = 3$ (a) shows that it is possible to limit total infections below $0.5 \%$ of total population size while keeping isolation person-time below $0.5 \%$ of total person-time (e.g., solutions between B and C), specifically with a testing and isolation scenario characterized by intermediate-to-high sensitivity ($\alpha _{\textrm{TP}} > 0.87$), relatively short duration of isolation ($5< 1 / \theta < 9$ days, centered around the assumed average recovery period of $1 / \gamma = 7$ days), almost daily testing of infected (that is, symptomatic, in our framework) individuals ($\kappa \approx 0.99$ days$^{-1}$), and essentially no testing for the non-infected ($\epsilon \approx 10^{-3}$ days$^{-1}$, b–c). Achieving even lower case counts (e.g., solutions between A and C in panel a) would lead to sensibly higher isolation person-time (up to $\approx 15 \%$ of total person-time), and would be associated with higher testing sensitivity ($\alpha _{\textrm{TP}} \rightarrow 1$) and longer isolation duration (e.g., $\approx 15$ days, b), with only minor quantitative differences in terms of testing rates (c). The Pareto front obtained for $\mathcal {R}_0 = 6$ (d) shows instead that, for instance, achieving a case count below $1 \%$ of total population size with isolation person-time below $1 \%$ of total person-time (solutions around C, which are the closest to the ideal point, a typically non-admissible solution in which each objective is independently optimized) would require high sensitivity ($\alpha _{\textrm{TP}} > 0.99$), a duration of isolation mandates that slightly exceeds the average recovery time ($1 / \theta \approx 10$ days), almost daily testing for infected (i.e., symptomatic) individuals ($\kappa \rightarrow 1$ days$^{-1}$), and essentially no testing for the non-infected ($\epsilon \rightarrow 10^{-3}$ days$^{-1}$, e–f).

## Discussion

In this paper, we have addressed the opportunities and challenges posed by testing, complemented by mandatory isolation for individuals classified as infected, as a tool for limiting the transmission of an infectious disease. Epidemic management may in general require seeking trade-off solutions to try and balance the reduction of the health-related burden of disease, on the one hand, and negative socioeconomic impact, on the other (Lasaulce et al. 2021; Ash et al. 2022). This is especially true in the presence of an emerging pathogen (Morens and Fauci 2020), for which vaccines or specific medical treatment may not be readily available, and which must thus be primarily contrasted with various non-pharmaceutical interventions—which, in some cases, may even include general lockdowns, as shown by the responses set-up by governments around the world to contrast the COVID-19 pandemic (Hale et al. 2021). The extraordinary socioeconomic cost of such society-wide measures makes individually-focused solutions, like surveillance testing coupled with mandatory isolation for infected people, clearly appealing. Testing, however, is by its own nature imperfect: when it lets infected individuals go undiagnosed (false negatives), it contributes to further the spread of disease; when it misdiagnoses non-infected individuals as infected (false positives), it produces an unnecessary socioeconomic burden (Gray et al. 2020).

To analyze the trade-offs specifically imposed by testing and mandatory isolation on the containment of an epidemic outbreak, we have extended the classic SIR model, in which the population is subdivided into homogeneous epidemiological groups (susceptible, infected, and recovered people), to account for an additional stratification based on the outcomes of testing (people who never tested positive, tested positive and are currently isolated, tested negative after isolation). Using this model, we showed that diagnostic testing and mandatory isolation can represent effective tools for epidemic containment, at least if $\mathcal {R}_0$ values are not too high. This result, in turn, suggests that these tools might work best when coupled with other containment measures aimed at reducing pathogen transmission. We also showed that testing and isolation remain effective even in the presence of false negative and false positive results, with the somehow expected corollary that more accurate testing reduces the effort required to curb transmission. By using case count and isolation person-time as key performance indicators, we explored the epidemiological and socioeconomic impacts of a wide range of testing and isolation scenarios. In some cases, we found nontrivial links between the testing parameters and the selected indicators, in a way echoing previous research reporting non-monotonic relationships between, e.g., testing rates and the effectiveness of testing and isolation as epidemic control tools (see, for instance, Gharouni et al. 2022; Zhang and Britton 2022).

Concerning the identification of efficient testing and isolation scenarios from a multi-criterial perspective, we found that the combination of frequent, high-sensitivity testing of infected (i.e., symptomatic, in our model) individuals and mandatory isolation lasting slightly longer than the average recovery time from infection may strike a reasonable balance between the health-related and the socioeconomic burden of disease. However, we remark that suggesting a specific solution goes beyond the scope of our modeling approach, as an informed decision-maker should instead be entrusted with the task of peaking among (ideally, Pareto-efficient) alternative solutions (Ehrgott 2005). It is also to be noted that the testing and isolation scenarios identified as Pareto-efficient in our analysis may depend upon modeling assumptions about the dynamics of disease transmission (e.g., frequency- vs. density-dependent force of infection), the implementation details of testing (e.g., whether testing capacity is limited, or whether contact tracing is enforced; see, for instance, Grassly et al. 2020; Baik et al. 2022; Zhang and Britton 2022), the possible behavioral responses of the population (e.g., whether individuals self-reduce their potential exposure as a result of perceived infection risk, or whether they fully comply with isolation orders; see, for example, Betsch et al. 2021; Bevan et al. 2021), and/or the choice of indicators used to weigh pros and cons of testing and isolation (e.g., *avoided* cases and/or *superfluous* isolation person-time, just to mention small, yet significant variations of the objective functions considered here).

Some of the alternative assumptions just exemplified are studied in Appendix S1 (Supplementary Information). To summarize the main results of the sensitivity analysis reported there, we can say that the findings presented in this work seem to be robust to a switch from frequency- to density-dependent contacts (Figure S1; note, however, that this result may depend upon the parameterization of the model), quite heavily influenced by some features of testing (namely, by strong limitations to the capacity of the testing infrastructure, Figure S2a–b) but not so much by others (such as the implementation of contact tracing, Figure S2c–d; note, however, that this results might be linked to the simplistic structure of the SIR model), and also remarkably impacted by individuals’ behavior (in particular, by the self-avoidance of exposure-prone activities, Figure S2e–f, and the unwillingness to comply with testing policies, Figure S2g–h). Perhaps unsurprisingly, among the alternative model formulations explored in Appendix S1, those showing the strongest deviations from the results presented here entail profound modifications to the formulation of the force of infection (like in the case of changes in the behavior of the population) or to the structure of the testing system (like in the case of a limited testing capacity)—two cornerstones of model (1).

In addition to considering some of the alternative hypotheses mentioned above, our model could be usefully extended in several other directions:

- it could be made more disease-specific, namely by modifying the standard SIR model to better describe the peculiarities of the transmission cycle of a given pathogen, e.g., following the modeling approach already proposed by Baik et al. (2022) for COVID-19. As an example, the SIR model has been extended to include exposed (infected but not yet infectious) individuals, as well as pre-symptomatic and asymptomatic infectious individuals, in order to more closely describe the transmission routes of the SARS-CoV-2 virus (Gatto et al. 2020). Such extension would also allow considering the relationship between viral dynamics and test accuracy, namely by accounting for different sensitivity and specificity values for individuals in different epidemiological compartments, as discussed by Mercer and Salit (2021) for COVID-19. Potential applications are not just limited to the SARS-CoV-2 pandemic, though, as testing for other infectious agents may induce trade-offs that are not unlike the ones we have discussed here. This is the case, for instance, of tuberculosis: on the one hand, identifying missed cases is of paramount importance to prevent further spread; on the other, false positives may lead to heavy consequences for single individuals (unneeded treatment), families (income loss), and society as a whole (ineffective resource allocation and disease surveillance; Houben et al. 2019);
- it could be extended to account for the simultaneous use of a mix of testing tools, instead of just one as implicitly assumed here. During a large-scale epidemic, in fact, different tests may be developed and commercialized, in particular for point-of-care or self-diagnosis use. Such tests can have widely ranging overall performances: as an example, a review of commercial lateral flow devices for detecting SARS-CoV-2 found 38–99 $\%$ sensitivity and 92–100 $\%$ specificity ranges (Mistry et al. 2021; note that these figures should be interpreted with caution because the gold-standard test based on reverse transcriptase polymerase chain reaction used to assess the performance of lateral flow devices is not perfect per se, as shown, e.g., by Kucirka et al. 2020). While we maintain that a negative relationship between true negative and true positive rates will be found at the population level even with a mix of different tests, relaxing eqn. (3) might still have nontrivial epidemiological and socioeconomic implications;
- it could accommodate time-varying testing and isolation protocols, following the observation that the enforcement of these tools may vary during the course of a large-scale epidemic (Brauner et al. 2021). In this case, optimal control theory could be used to design adaptive testing and isolation policies (Lenhart and Workman 2007);
- it could be extended to include other measures for epidemic containment, in addition to testing and isolation, in order to discuss possible trade-offs emerging from the simultaneous applications of multiple controls, as typically done in more realistic settings (Flaxman et al. 2020; Hsiang et al. 2020; Choi and Shim 2021; Mari et al. 2021);
- it could account for a larger set of epidemiological and socioeconomic indicators. While the metrics considered here or the alternative ones introduced in Appendix S1 (avoided cases and superfluous isolation person-time, Figures S3 and S4) could be evaluated using the basic formulation of model (1), an effective description of others (e.g., hospitalizations or deaths, life-years lost or health-adjusted life years, superfluous quarantines, avoided business or school closure, gross domestic product loss) might require a model with a more complicated structure (for instance, with more epidemiological compartments, and/or including an age-based or socioeconomic stratification). Expanding the range of the indicators considered in our multi-criterial analysis could remarkably improve its realism, and allow us to better meet the complexity of actual epidemic management and the challenges associated with a thorough evaluation of the multi-dimensional impacts of an epidemic (Chen et al. 2021; Igoe et al. 2023).

We believe that, despite its simplicity (or, perhaps, because of it), our approach allows us to effectively address the intrinsically multi-criterial nature of decision-making in public health. We emphasize that our results should not be seen as a justification for healthcare rationing (i.e., finding the best allocation of possibly insufficient healthcare resources, another theme that has widely been discussed since the beginning of the COVID-19 pandemic, see Emanuel et al. 2020; White and Lo 2020), rather as a call for careful assessment and efficient design of epidemic containment measures that explicitly acknowledge the unavoidable conflicts emerging when multiple objectives are involved, especially in the presence of imperfect control tools.

## Supplementary Information

Below is the link to the electronic supplementary material.

Supplementary file 1 (pdf 1076 KB)

## Data Availability

This work has no associated data.

## Code Availability

The code to simulate the SIR-like model extended to account for the effects of imperfect testing is available at https://github.com/lorenzo-mari/SIR-testing.
