# Supplementary material for: Epidemic Management via Imperfect Testing: A Multi-criterial Perspective
Source: Bull Math Biol. 2023 Jun 9;85(7):66. doi: 10.1007/s11538-023-01172-1 (PMC10255952; doi:10.1007/s11538-023-01172-1)
Supplement: Supplementary file 1 — (pdf 1076 KB) [file 11538_2023_1172_MOESM1_ESM.pdf]

Epidemic management via imperfect testing: A multi-criterial perspective
Supplementary Information

Giuseppe Palma[1], Damiano Caprioli[2] and Lorenzo Mari[3*]

[1]Institute of Nanotechnology, National Research Council, Campus Ecotekne, Via Monteroni, Lecce, 73100, LE, Italy.
[2]Department of Astronomy & Astrophysics, E. Fermi Institute, University of Chicago, 5640 South Ellis Avenue, Chicago, 60637, IL, United States.
[3]Dipartimento di Elettronica, Informazione e Bioingegneria, Politecnico di Milano, Via Ponzio 34/5, Milano, 20133, MI, Italy.

*Corresponding author(s). E-mail(s): lorenzo.mari@polimi.it; Contributing authors: giuseppe.palma@cnr.it; caprioli@uchicago.edu;

S1 Alternative problem formulations

In addition to the model formulation presented in the main text, here we explore some alternative hypotheses concerning the dynamics of disease transmission (density-dependent transmission), the way in which testing is implemented (limited testing capacity, contact tracing), and possible behavioral responses of the population (self-induced exposure avoidance, limited isolation compliance). We also investigate the use of alternative performance indicators to evaluate the epidemiological and socioeconomic impacts of epidemic control (case-count reduction compared to a no-control scenario, superfluous isolation person-time).

S1.1 Density-dependent transmission

While model (1) in the main text includes frequency-dependent transmission, an alternative assumption that is commonly made when describing the spread

of human pathogens is that the contact rate depends upon the density of the population. In this case, the force of infection is proportional to the abundance of infected individuals (akin to the law of mass action in chemistry). Model (1) in the main text can thus be reformulated as

$$\begin{aligned}
\dot{S}_n &= -(\lambda_d + \epsilon\alpha_{\mathrm{FP}})S_n \\
\dot{S}_p &= \epsilon\alpha_{\mathrm{FP}}S_n - \theta\alpha_{\mathrm{TN}}S_p \\
\dot{S}_c &= \theta\alpha_{\mathrm{TN}}S_p - \lambda_d S_c \\
\dot{I}_n &= \lambda_d S_n - (\gamma + \kappa\alpha_{\mathrm{TP}})I_n \\
\dot{I}_p &= \kappa\alpha_{\mathrm{TP}}I_n - (\gamma + \theta\alpha_{\mathrm{FN}})I_p \\
\dot{I}_c &= \lambda_d S_c + \theta\alpha_{\mathrm{FN}}I_p - \gamma I_c \\
\dot{R}_n &= \gamma I_n - \epsilon\alpha_{\mathrm{FP}}R_n \\
\dot{R}_p &= \gamma I_p + \epsilon\alpha_{\mathrm{FP}}R_n - \theta\alpha_{\mathrm{TN}}R_p \\
\dot{R}_c &= \gamma I_c + \theta\alpha_{\mathrm{TN}}R_p \,,
\end{aligned} \tag{S1}$$

where

$$\lambda_d = \beta_d(I_n + I_c) \tag{S2}$$

is the density-dependent force of infection and β_d is the density-dependent transmission rate.

A sensitivity analysis of the model results with respect to its main parameters is shown in Figure S1. Comparing this figure to its counterpart for frequency-dependent transmission (Figure 4 in the main text) shows that, although some differences emerge, they are relatively small and mostly quantitative. Therefore, for the range of parameters explored in our analysis, assuming frequency- or density-dependent infection dynamics does not substantially alter either the overall epidemiological picture or the essence of the results presented in the main text. The only qualitative discrepancy between the results presented in Figure S1 and Figure 4 in the main text may be represented by the increase in case count observed for high values of ϵ, which is much less pronounced with density-dependent transmission than it is with frequency-dependent transmission. This difference is essentially due to the fact that the nonlinear feedback discussed in the main text for frequency-dependent contacts is missing in the presence of a density-dependent force of infection. From a quantitative perspective, instead, we note that the robustness of the presented results might also be at least partially due to the chosen parameterization of the model. As shown in the baseline simulation of the model with frequency-dependent contacts (Figure 3 in the main text), the share of individuals who are subject to an isolation mandate reaches a maximum value of $\approx 7\%$. Therefore, even at the peak of the isolation prevalence curve, the denominator of equation (2) in the main text is $\approx 0.93N$. Parameterizations describing more aggressive isolation policies might result in larger quantitative differences between model simulations that assume either frequency- or density-dependent transmission.

S1.2 Limited testing capacity

The testing rates in model (1) in the main text are assumed to be constant, i.e., independent of the number of tests that are administered daily. However, with a constant testing rate, this quantity may change considerably during the course of an epidemic, following the temporal dynamics of the various epidemiological compartments. In particular, when disease prevalence is high (or when a large fraction of non-infected gets tested), the daily number of required tests might exceed resource availability or the capacity of the testing infrastructure. It thus seems reasonable to hypothesize that testing rates effectively decrease (or, equivalently, that the waiting time before getting access to testing increases) for increasing demand for testing. Model (1) in the main text can thus be reformulated as

$$\begin{aligned}
\dot{S}_n &= -(\lambda + \epsilon\phi(\mathcal{T})\alpha_{\mathrm{FP}})S_n \\
\dot{S}_p &= \epsilon\phi(\mathcal{T})\alpha_{\mathrm{FP}}S_n - \theta\phi(\mathcal{T})\alpha_{\mathrm{TN}}S_p \\
\dot{S}_c &= \theta\phi(\mathcal{T})\alpha_{\mathrm{TN}}S_p - \lambda S_c \\
\dot{I}_n &= \lambda S_n - (\gamma + \kappa\phi(\mathcal{T})\alpha_{\mathrm{TP}})I_n \\
\dot{I}_p &= \kappa\phi(\mathcal{T})\alpha_{\mathrm{TP}}I_n - (\gamma + \theta\phi(\mathcal{T})\alpha_{\mathrm{FN}})I_p \\
\dot{I}_c &= \lambda S_c + \theta\phi(\mathcal{T})\alpha_{\mathrm{FN}}I_p - \gamma I_c \\
\dot{R}_n &= \gamma I_n - \epsilon\phi(\mathcal{T})\alpha_{\mathrm{FP}}R_n \\
\dot{R}_p &= \gamma I_p + \epsilon\phi(\mathcal{T})\alpha_{\mathrm{FP}}R_n - \theta\phi(\mathcal{T})\alpha_{\mathrm{TN}}R_p \\
\dot{R}_c &= \gamma I_c + \theta\phi(\mathcal{T})\alpha_{\mathrm{TN}}R_p \, ,
\end{aligned} \tag{S3}$$

where

$$\mathcal{T} = \frac{\epsilon(S_n + R_n) + \kappa I_n + \theta(S_p + I_p + R_p)}{S_n + I_n + R_n + S_p + I_p + R_p}$$

is the potential, per-capita demand for testing and

$$\phi(\mathcal{T}) = \frac{1}{1 + \left(\frac{\mathcal{T}}{\eta}\right)^2}$$

is a monotonically decreasing function assumed to modulate the baseline contact rates ϵ and κ. Note, in particular, that $\phi(\mathcal{T}) = 1/2$ if $\mathcal{T} = \eta$; therefore, η can be thought of as the half-saturation constant of the testing system.

Figure S2a–b shows the effects of different values of the half-saturation constant η on case count and isolation person-time. Clearly, for large values of η (say, $\eta > 1$), the results obtained with model (S3) converge to those of model (1) in the main text. On the other hand, for smaller values of η, the saturation of the testing system may considerably reduce the effectiveness of epidemic control, thus leading to larger case counts independently of the transmissibility of the pathogen (a). In these cases, isolation person-time is

also reduced, although local maxima can be present for intermediate values of η with pathogens characterized by relatively low transmission potential (b).

We also note that, in case of limited testing capacity, a prioritization strategy to decide who gets actually tested (or tested first) may be implemented. For instance, precedence could be given to strongly symptomatic individuals or those who belong to high-risk groups within the population (e.g., healthcare providers), while individuals with no clinical symptoms or belonging to lower-risk categories could be tested only if the demand for testing has not maxed out yet.

S1.3 Contact tracing

In addition to the case considered in the previous section, another situation in which testing rates may non-linearly depend upon the state of the system occurs if the close contacts of positive test recipients are actively traced. Depending on the timescales involved in the dynamics of infection, these contacts could be either preventively quarantined, or immediately administered a test to ascertain their infection status and placed in isolation in case they test positive. For the sake of simplicity (i.e., to avoid the definition of new state variables), we use the latter version of contact tracing and reformulate model (1) in the main text as

$$
\begin{aligned}
\dot{S}_n &= -[\lambda + (\epsilon + \sigma(\mathcal{P}))\alpha_{\mathrm{FP}}]S_n \\
\dot{S}_p &= (\epsilon + \sigma(\mathcal{P}))\alpha_{\mathrm{FP}}S_n - \theta\alpha_{\mathrm{TN}}S_p \\
\dot{S}_c &= \theta\alpha_{\mathrm{TN}}S_p - \lambda S_c \\
\dot{I}_n &= \lambda S_n - [\gamma + (\kappa + \sigma(\mathcal{P}))\alpha_{\mathrm{TP}}]I_n \\
\dot{I}_p &= (\kappa + \sigma(\mathcal{P}))\alpha_{\mathrm{TP}}I_n - (\gamma + \theta\alpha_{\mathrm{FN}})I_p \\
\dot{I}_c &= \lambda S_c + \theta\alpha_{\mathrm{FN}}I_p - \gamma I_c \\
\dot{R}_n &= \gamma I_n - (\epsilon + \sigma(\mathcal{P}))\alpha_{\mathrm{FP}}R_n \\
\dot{R}_p &= \gamma I_p + (\epsilon + \sigma(\mathcal{P}))\alpha_{\mathrm{FP}}R_n - \theta\alpha_{\mathrm{TN}}R_p \\
\dot{R}_c &= \gamma I_c + \theta\alpha_{\mathrm{TN}}R_p \,,
\end{aligned}
\tag{S4}
$$

where

$$\mathcal{P} = \epsilon\alpha_{\mathrm{FP}}(S_n + R_n) + \kappa\alpha_{\mathrm{TP}}I_n$$

is the rate of positive case discovery and

$$\sigma(\mathcal{P}) = \frac{\xi\mathcal{P}}{S_n + I_n + R_n}$$

is the additional per-capita testing rate induced by contact-tracing activities, assumed to be proportional to the average number of traced contacts per positive case, ξ, and to apply equally to all untested individuals independently of

their actual infection status. Note that the assumption of a well-mixed community in model (S4) does not allow a more realistic description of the social interaction networks of the newly discovered positive cases, whose investigation instead plays a significant role in real-world contact tracing efforts.

Figure S2c–d shows that, for a pathogen with relatively low transmission potential, an increasing contact-tracing effort is linked to a decreasing case count; for a more transmissible pathogen, instead, there might even exist an optimal contact-tracing effort to minimize case count (c). In both cases, however, the effect of our simple implementation of contact tracing on case count is modest at best. Stronger tracing efforts are also linked to a progressive increase in isolation person-time (d).

While the performances of contact tracing just observed may seem underwhelming, we note that contact tracing is expected to be most successful at limiting disease spread if a significant delay exists between exposure to the infectious agent and infectiousness onset, especially if the close contacts of the recipient of a positive test are asked to preventively quarantine for a few days before taking a test. Another case in which contact tracing would likely prove effective is the presence of asymptomatic infections. In these cases, contact tracing may allow an early removal from the general community of individuals who have already been infected but are not infectious yet (exposed or pre-symptomatic individuals), or are covertly transmitting the disease (asymptomatic individuals), obviously provided that testing is effective also at identifying these latent stages of the infection.

S1.4 Behavioral change

One basic assumption of model (1) in the main text is that the transmission rate β is independent of the temporal dynamics of the outbreak. However, it may be reasonable to assume that the individuals might virtuously adapt their behavior as a response to an unfolding epidemic, namely by limiting those activities that might increase their chances to be exposed to the pathogen. A simple way to account for this phenomenon is to reformulate model (1) in the main text as

$$\begin{aligned}
\dot{S}_n &= -(\lambda_v + \epsilon\alpha_{\mathrm{FP}})S_n \\
\dot{S}_p &= \epsilon\alpha_{\mathrm{FP}}S_n - \theta\alpha_{\mathrm{TN}}S_p \\
\dot{S}_c &= \theta\alpha_{\mathrm{TN}}S_p - \lambda_v S_c \\
\dot{I}_n &= \lambda_v S_n - (\gamma + \kappa\alpha_{\mathrm{TP}})I_n \\
\dot{I}_p &= \kappa\alpha_{\mathrm{TP}}I_n - (\gamma + \theta\alpha_{\mathrm{FN}})I_p \\
\dot{I}_c &= \lambda_v S_c + \theta\alpha_{\mathrm{FN}}I_p - \gamma I_c \\
\dot{R}_n &= \gamma I_n - \epsilon\alpha_{\mathrm{FP}}R_n \\
\dot{R}_p &= \gamma I_p + \epsilon\alpha_{\mathrm{FP}}R_n - \theta\alpha_{\mathrm{TN}}R_p \\
\dot{R}_c &= \gamma I_c + \theta\alpha_{\mathrm{TN}}R_p \,,
\end{aligned} \tag{S5}$$

where

$$\lambda_v = \lambda\psi(\mathcal{Q}) \tag{S6}$$

is the behavior-dependent force of infection, with

$$\mathcal{Q} = \frac{\mathcal{P}}{S_n + I_n + R_n}$$

being the per-capita rate of positive case discovery and

$$\psi(\mathcal{Q}) = \frac{1}{1 + \left(\frac{\mathcal{Q}}{\chi}\right)^2}$$

being a monotonically decreasing function assumed to modulate the baseline force of infection λ. The parameter χ can be thought of as a half-saturation constant, as $\psi(\mathcal{Q}) = 1/2$ if $\mathcal{Q} = \chi$.

The effects of this behavioral adaptation on case count and isolation person-time are shown in Figure S2e–f. Similarly to what was observed in panels a–b, for large values of χ (say, $\chi > 1$), the results obtained with model (S5) converge to those of model (1) in the main text. On the other hand, for smaller values of the half-saturation constant, decreasing values of χ (corresponding to a stronger behavioral response) are associated with lower case count (e) and isolation person-time (f). These results clearly point to the importance of virtuous individual responses in alleviating the trade-offs between the necessity of effective disease transmission control, on one hand, and the need to preserve socioeconomic welfare, on the other.

We note that behavioral adaptations related to testing may not always yield positive impacts in terms of transmission containment and socioeconomic burden. As an example, the willingness of the population to subject themselves to testing (possibly followed by mandatory isolation) might decrease over time, e.g., in response to reduced perception of infection risk or increased socioeconomic stress, thereby affecting disease surveillance and possibly reducing the expected positive outcomes of the testing campaign.

S1.5 Limited compliance with isolation orders

Model (1) in the main text assumes that individuals who test positive for the pathogen are subject to mandatory isolation and that they are fully compliant with this prescription. However, it is not unrealistic to imagine that at least some recipients of an isolation mandate might unlawfully try and engage in social activities, thereby contributing to community transmission (if infected) or being exposed to it (if susceptible). To take limited compliance with mandatory isolation under consideration, model (1) in the main text can

be reformulated as

$$\begin{aligned}
\dot{S}_n &= -(\lambda_l + \epsilon\alpha_{\mathrm{FP}})S_n \\
\dot{S}_p &= \epsilon\alpha_{\mathrm{FP}}S_n - (\zeta\lambda_l + \theta)\alpha_{\mathrm{TN}}S_p \\
\dot{S}_c &= \theta\alpha_{\mathrm{TN}}S_p - \lambda_l S_c \\
\dot{I}_n &= \lambda_l S_n - (\gamma + \kappa\alpha_{\mathrm{TP}})I_n \\
\dot{I}_p &= \zeta\lambda_l S_p + \kappa\alpha_{\mathrm{TP}}I_n - (\gamma + \theta\alpha_{\mathrm{FN}})I_p \\
\dot{I}_c &= \lambda_l S_c + \theta\alpha_{\mathrm{FN}}I_p - \gamma I_c \\
\dot{R}_n &= \gamma I_n - \epsilon\alpha_{\mathrm{FP}}R_n \\
\dot{R}_p &= \gamma I_p + \epsilon\alpha_{\mathrm{FP}}R_n - \theta\alpha_{\mathrm{TN}}R_p \\
\dot{R}_c &= \gamma I_c + \theta\alpha_{\mathrm{TN}}R_p\,,
\end{aligned} \tag{S7}$$

with ζ being the fraction of non-complying recipients of isolation orders and

$$\lambda_l = \beta\frac{I_n + I_c + \zeta I_p}{S_n + S_c + I_n + I_c + R_n + R_c + \zeta(S_p + I_p + R_p)} \tag{S8}$$

being the resulting force of infection (note the changes at both the numerator and the denominator of the expression), assumed to be frequency dependent like in model (1) in the main text.

The epidemiological and socioeconomic implications of limited isolation compliance are shown in Figure S2g–h. Quite unsurprisingly, higher non-compliance is associated with increasing case count (g) and isolation person-time (h). These results further stress the importance of correct individual behavior for the effective management of an ongoing epidemic outbreak.

Symmetrically to what was discussed in the previous section about a possible reduction of the willingness to be tested, individuals might also adapt their behavior in a somewhat opposite direction, namely that of voluntarily self-isolating. This response, which may grow stronger if the fear of infection increases over the course of an outbreak, could have positive implications for epidemic control, but might also exacerbate the socioeconomic burden of disease.

S1.6 Alternative performance indicators

The choice of performance indicators made in the main text to assess the effects of testing and isolation as means to manage an unfolding epidemic is certainly not unique. Here, we repeat the sensitivity analysis and multi-criterial assessment presented in Figures 4 and 5 in the main text using a pair of different epidemiological and socioeconomic indicators, namely:

- the relative reduction in case count projected by the model with testing and isolation as compared to a no-control scenario, and

- the share of superfluous isolation time, evaluated as a fraction of total person time.

Note that, although a pair of indicators is used again to make the results of multi-criterial assessment easier to visualize, nothing technically excludes the use of a larger set of performance indicators.

A sensitivity analysis of the model projections with respect to the two alternative performance indicators just introduced is shown in Figure S3. As did in the main text, a timespan of $t_\omega = 90$ days after the start of containment measures has been considered to assess the effectiveness of controls. In the presence of an aggressive pathogen (higher $\mathcal{R}_0$), higher values of test sensitivity are associated with a monotonically increasing effectiveness in reducing infections (a). With a less aggressive pathogen (lower $\mathcal{R}_0$), instead, the relationship between test sensitivity and case count reduction is monotonic only up to $\alpha_{\text{TP}} \approx 0.95$, after which the effectiveness of testing and isolation for infection control declines, reaching a local minimum for $\alpha_{\text{TP}} \approx 0.99$, before eventually increasing again and reaching $\approx 99\%$ for $\alpha_{\text{TP}} = 1$. The relationship between sensitivity and superfluous isolation person-time is not trivial either: for an aggressive pathogen, it is monotonically increasing; for a milder one, a local minimum is projected to occur for $\alpha_{\text{TP}} \approx 0.88$ (b). Longer isolation mandates determine an increase of testing effectiveness in reducing infections (c), but also an increase of superfluous isolation person-time (d), owing to recovered individuals waiting to be released. Higher testing rates for infected individuals also lead to stronger case count reduction (e). On the other hand, the relationship between the testing rate and superfluous isolation person-time is nontrivial, peaking at intermediate values of the rate in both of the considered $\mathcal{R}_0$ scenarios (f). Higher testing rates for non-infected individuals may lead to either higher or lower effectiveness in infection control depending on the value of $\mathcal{R}_0$: with a less aggressive pathogen, more frequent testing leads to smaller case count reduction; with a more aggressive one, higher testing rates determine progressively higher effectiveness in disease control (g). On the other hand, higher values of the testing rate for non-infected individuals lead to increasing shares of superfluous isolation person-time (h).

Figure S4 shows the Pareto fronts and the corresponding Pareto-efficient solutions evaluated over the same parameter ranges explored in Figure S3. Note that, in this case, the health-related objective has to be maximized rather than minimized. The Pareto front obtained for $\mathcal{R}_0 = 3$ (a) shows that it is possible to achieve $\gg 99\%$ infection reduction while limiting superfluous isolation time to $\ll 1\%$ (e.g., solutions between B and C), specifically with testing and isolation scenarios characterized by intermediate sensitivity ($0.87 < \alpha_{\text{TP}} < 0.97$), relatively short isolation duration ($5 < 1/\theta < 7$ days), almost daily testing of infected (thus, symptomatic, in our framework) individuals ($\kappa \to 1$ days^{-1}), and essentially no testing for the non-infected ($\epsilon \to 10^{-3}$ days^{-1}, b–c). Achieving a further reduction of case counts (e.g., solutions between A and C in panel a) would lead to sensibly higher superfluous isolation time (up to $\approx 3\%$ of total person-time), and would be associated with higher testing sensitivity

($\alpha_{\mathrm{TP}} > 0.97$) and longer isolation duration ($7 < 1/\theta < 18$ days, b), with only minor quantitative differences in terms of testing rates (c). The Pareto front obtained for $\mathcal{R}_0 = 6$ (d) shows instead that achieving $\approx 99\%$ infection reduction with at most 1% superfluous isolation time (e.g., solutions around C) would require high sensitivity ($\alpha_{\mathrm{TP}} \approx 0.98$), isolation with a duration similar to the recovery period from infection ($1/\theta \approx 7$ days), and, again, almost daily testing for infected (hence, symptomatic, in our model), and essentially no testing for non-infected individuals ($\kappa \to 1$ days^{-1}, $\epsilon \to 10^{-3}$ days^{-1}, e–f).

Supplementary Figures

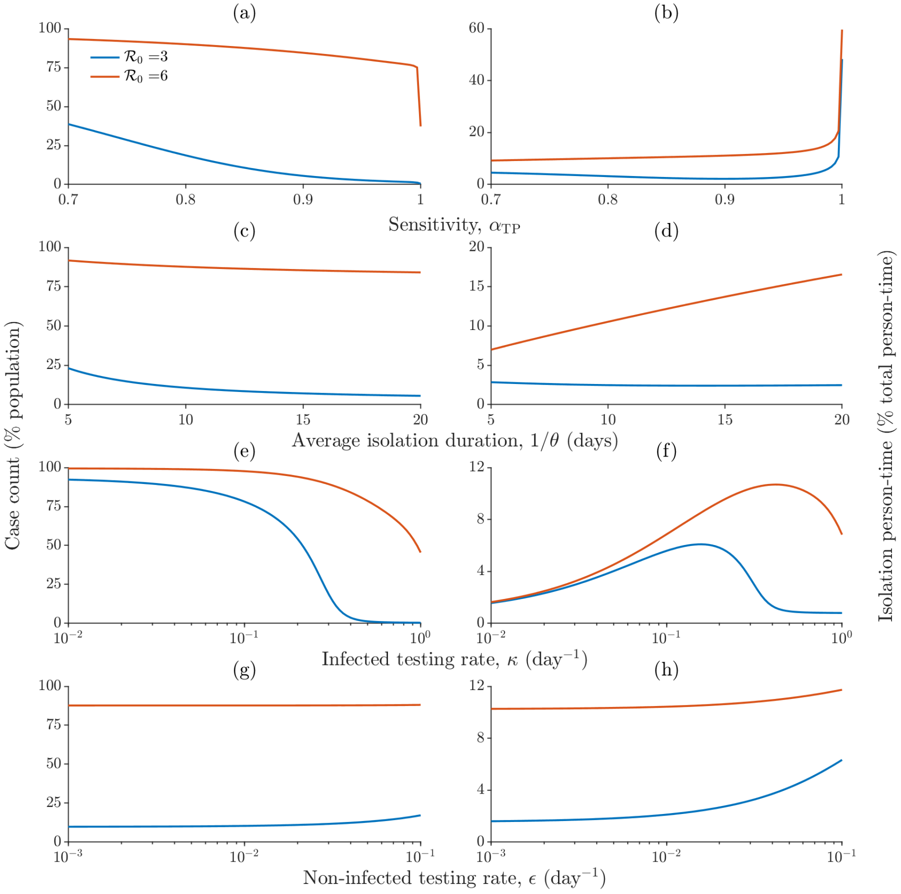

Fig. S1 Sensitivity analysis of model (S1), accounting for a density-dependent force of infection, with respect to variations of testing and isolation parameters. Details as in Figure 4 in the main text, except for the transmission rate $\beta_d = \beta/N = (\mathcal{R}_0\gamma)/N$, which has been chosen so that $\lambda_d \equiv \lambda$ in a neighborhood of the disease-free equilibrium.

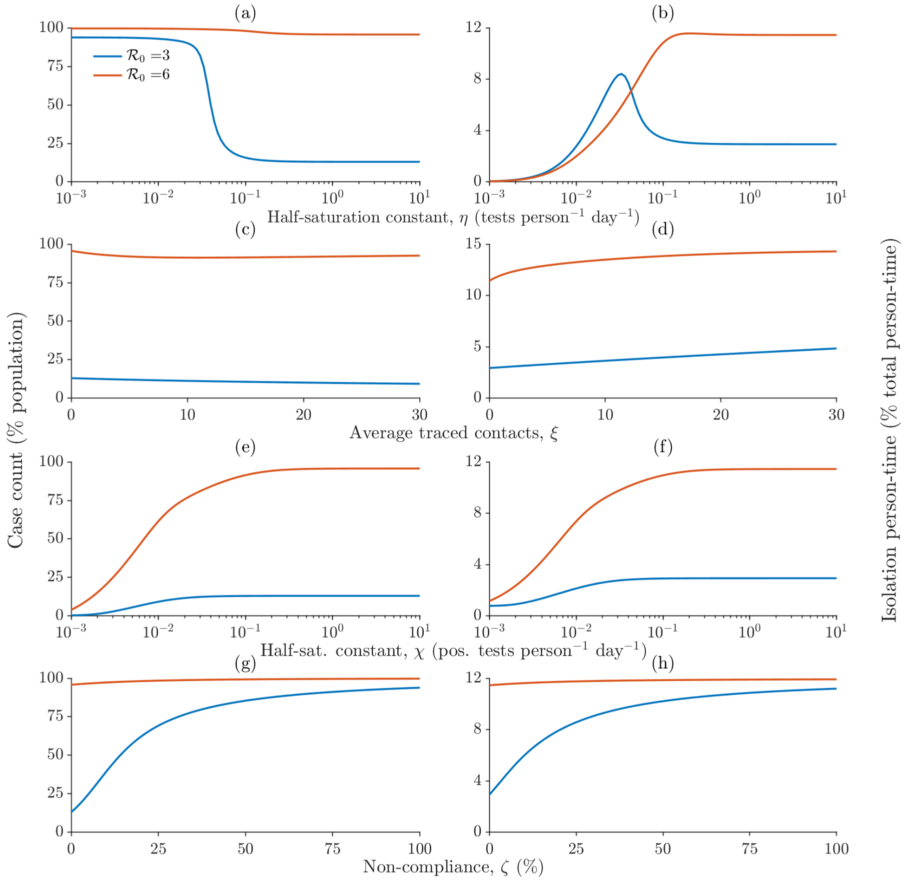

Fig. S2 Sensitivity analysis of alternative model formulations (S3), (S4), (S5), and (S7). (a–b) Effect of the half-saturation constant η of testing capacity (model (S3)) on case count (a) and isolation person-time (b). (c–h) As in a–b, with respect to the average number α of traced contacts (model (S4), c–d), the half-saturation constant χ of the behavioral response to the case discovery rate (model (S5), e–f), and the fraction ζ of individuals who are not compliant with isolation mandates (model (S7), g–h). Other details as in Figure 4 in the main text.

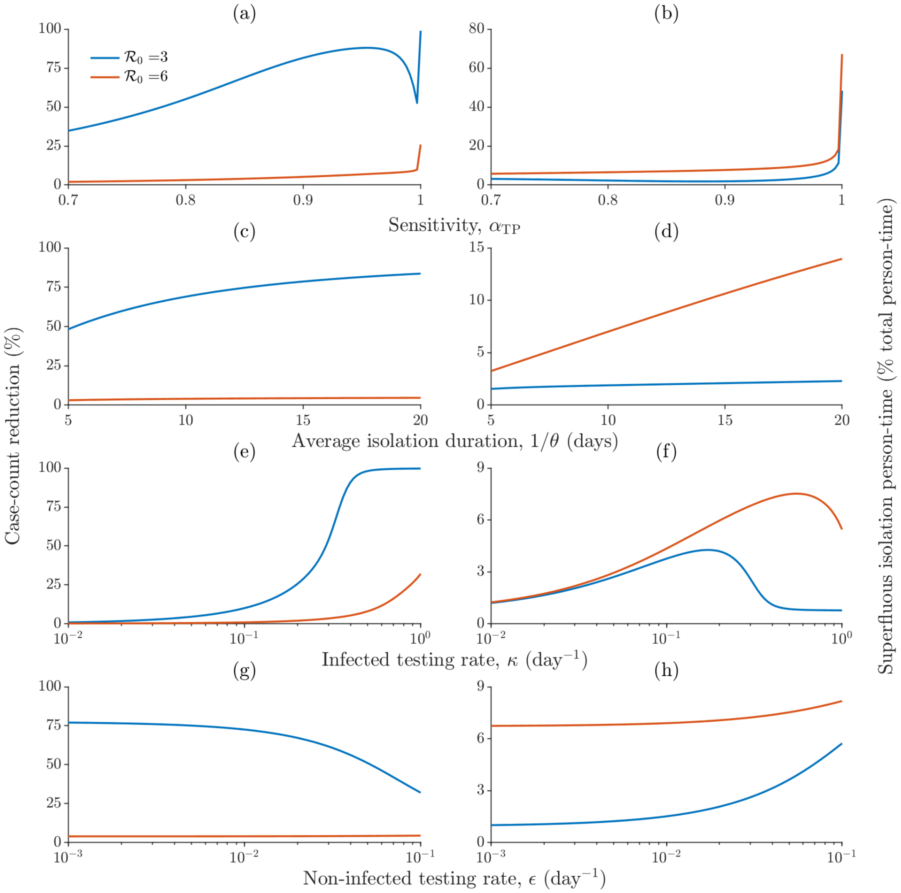

Fig. S3 Sensitivity analysis of model (1) in the main text with respect to variations of testing and isolation parameters, using case-count reduction and superfluous isolation person-time as alternative performance indicators. Other details as in Figure 4 in the main text.

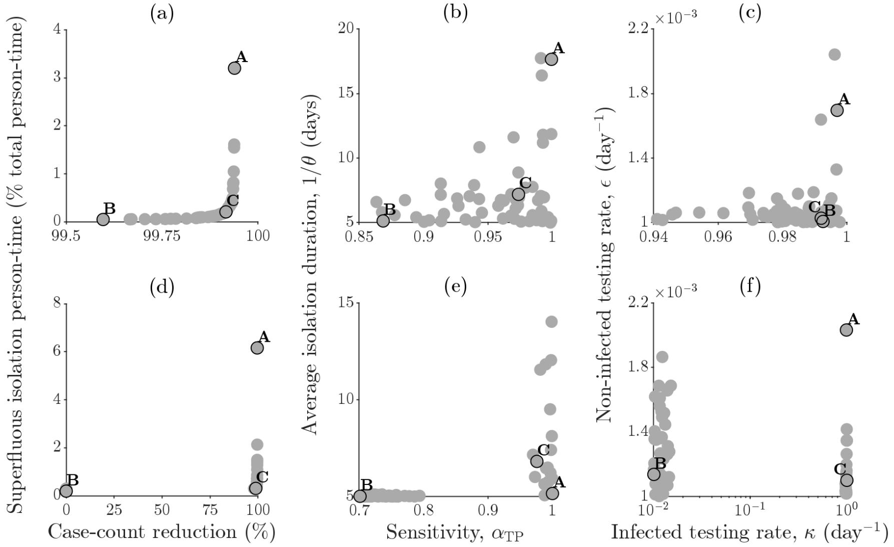

Fig. S4 Pareto-efficient scenarios for epidemic control, evaluated with case-count reduction and superfluous isolation person-time as the objective functions to be maximized and minimized, respectively. (a) Pareto front obtained for various combinations of test sensitivity, isolation duration, and testing frequency for infected and non-infected individuals, assuming $\mathcal{R}_0 = 3$. (b–c) Two possible projections of the four-dimensional set of Pareto-efficient solutions. (d–f) As in a–c, assuming $\mathcal{R}_0 = 6$. Testing and isolation scenarios have been obtained via Latin hypercube sampling (10^6 samples) of the parameter space explored in Figure S3. The points marked as A and B correspond to the solutions with maximum or minimum case count reduction among the explored alternatives, respectively, while C represents the solution that is closest to the ideal point. Other details as in Figure 5 in the main text.
